# The involvement of dityrosine crosslinking in α-synuclein assembly and deposition in Lewy Bodies in Parkinson’s disease

**DOI:** 10.1038/srep39171

**Published:** 2016-12-16

**Authors:** Youssra K. Al-Hilaly, Luca Biasetti, Ben J. F. Blakeman, Saskia J. Pollack, Shahin Zibaee, Alaa Abdul-Sada, Julian R. Thorpe, Wei-Feng Xue, Louise C. Serpell

**Affiliations:** 1 School of Life Sciences, University of Sussex, Falmer, BN1 9QG, UK; 2 College of Sciences, Chemistry Department, Al-Mustansiriyah University, Baghdad, Iraq; 3 School of Biosciences, University of Kent, Canterbury, CT2 7NJ, UK; 4 Laboratory of Molecular Biology, MRC Centre, Hills Rd, Cambridge, CB2 OQH, UK.

## Abstract

Parkinson’s disease (PD) is characterized by intracellular, insoluble Lewy bodies composed of highly stable α-synuclein (α-syn) amyloid fibrils. α-synuclein is an intrinsically disordered protein that has the capacity to assemble to form β-sheet rich fibrils. Oxidiative stress and metal rich environments have been implicated in triggering assembly. Here, we have explored the composition of Lewy bodies in post-mortem tissue using electron microscopy and immunogold labeling and revealed dityrosine crosslinks in Lewy bodies in brain tissue from PD patients. *In vitro*, we show that dityrosine cross-links in α-syn are formed by covalent ortho-ortho coupling of two tyrosine residues under conditions of oxidative stress by fluorescence and confirmed using mass-spectrometry. A covalently cross-linked dimer isolated by SDS-PAGE and mass analysis showed that dityrosine dimer was formed via the coupling of Y39-Y39 to give a homo dimer peptide that may play a key role in formation of oligomeric and seeds for fibril formation. Atomic force microscopy analysis reveals that the covalent dityrosine contributes to the stabilization of α-syn assemblies. Thus, the presence of oxidative stress induced dityrosine could play an important role in assembly and toxicity of α-syn in PD.

Parkinson’s disease (PD) is the second most common neurodegenerative disease after Alzheimer’s disease (AD) in humans and both diseases are associated with the aggregation of proteins into amyloid deposits. PD is characterized by the formation of intracelluar amyloid fibril deposition of α-synuclein (α-syn) in Lewy bodies that accumulate in the substantia nigra of sufferers^1^. α-syn is an 140 amino-acid intrinsically disordered protein that can self-assemble to form β-sheet rich oligomers and amyloid fibrils^1^. It has been proposed that small early oligomeric forms of α-syn may lead to local degeneration of the dopaminergic cells of the substantia nigra^2^,^3^ and permeation of lipid bilayers^4^ whilst the fibrils themselves may cause structural damage to cellular membranes^5^.

Oxidative stress has been implicated in the pathogenesis of a number of neurodegenerative diseases, including AD and PD^6^,^7^. Oxidative stress can lead to a multitude of protein, lipid and nucleic acid damage and is implicated in the formation of dityrosine cross-links in the Amyloid-β (Aβ) peptide in amyloid plaques in AD^8^,^9^. Dityrosine cross-linking is formed by the ortho-ortho coupling of tyrosine residues and can take place intra- or inter-molecularly. Similar to Aβ, dityrosine cross-links can also be formed *in vitro* via metal catalyzed oxidation (MCO) of α-syn under physiological conditions^10^,^11^, and have been identified as biomarkers of oxidative stress in an 1-methyl-4-phenyl-1,2,3,6-tetrahydropyridine (MPTP) mouse model of PD^12^,^13^. Tyrosines are found at four positions in the α-syn sequence at residue 39, 125, 133 and 136 and previous work has highlighted the importance of Y39 in self-assembly^14^. Elevated levels of many metal ions such as iron and zinc have been reported in substantia nigra^15^,^16^. Studying the influence of dityrosine cross-links on α-syn conformation and the effects during amyloid formation will, therefore, provide insights into the importance of oxidative modification of tyrosine residues in the pathogenesis of PD.

Here, immunogold electron microscopy (EM) was used to examine the prevalence of dityrosines in Lewy bodies in PD brain tissue, and revealed the presence of dityrosine in α-syn in Lewy bodies. The ability of Cu^2+^ to promote the formation of *in vitro* dityrosine cross-linked α-syn was explored and the effect of the Cu^2+^ ion on α-syn amyloid self-assembly and conformation was investigated. The presence of dityrosine cross-links was examined using fluorescence spectroscopy, liquid chromatography electrospray ionization mass-spectrometry (LC-ESIMS/MS), SDS-PAGE, transmission electron microscopy (TEM), atomic force microscopy (AFM) and X-ray fibre diffraction (XRFD). Our results show that oxidative stress induces dityrosine cross-links, which could play an important role in assembly, stability and toxicity of α-syn in PD.

## Results

### Detection of dityrosine in Lewy bodies in brain sections from PD affected patients

**Figure 1:**
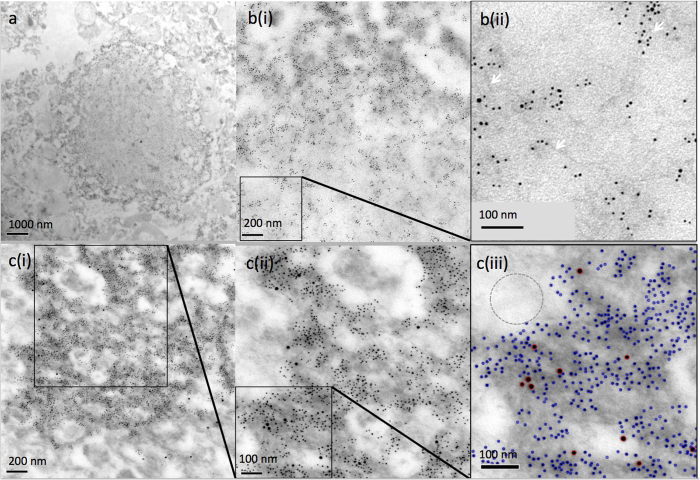
Immunogold labeling TEM within Lewy bodies from PD brains. (**a**) Shows a Lewy body taken from PD041 case (Table 1) and (**b**,**c**) show representative micrographs showing double labeling using the dityrosine antibody (15 nm) and anti-α-syn (5 nm) antibody on a PD substantia nigra brain section taken from PD028 case (Table 1) and independent areas at increasing magnifications. The micrographs reveal very dense anti-α-syn labeling of the Lewy bodies and confirm colocalization of dityrosine with α-syn within Lewy bodies. (**b**) (ii) reveals fibrillar structures labeled with both 5 and 15 nm gold particles supporting the view that dityrosine crosslinks are present within the fibrils in the Lewy bodies (white arrows). (**c**) (i, ii and iii) highlight these structures by increasing magnification to show the presence of both 5 and 15 nm gold labels and the 15 nm (red) and 5 nm (blue) have been highlighted in (**c**) (iii). An area outside of the Lewy body is highlighted (dotted circle) to show the absence of gold labeling.

Dityrosine cross-linking has been identified previously in α-syn and has been suggested to play important role in the pathogenesis of PD^17^,^18^. However, previous studies have not demonstrated direct physiological links between PD and dityrosine crosslinking. Here, post-mortem substantia nigra brain sections from PD patients were immunogold labeled using anti-dityrosine mouse monoclonal antibody and anti-α-syn within Lewy bodies in the brain sections and then visualized under TEM to explore the colocalization of gold-conjugated antibodies. Three cases were examined (see Table 1) and of these, two cases (PD028 and PD041) showed Lewy bodies which were examined further. The TEM images show the presence of dityrosine antibody labeling alongside extensive α-syn labeling in Lewy bodies (Fig. 1). In contrast, no labeling was observed for either dityrosine or α-syn outside of the Lewy bodies (circled in Fig. 1c(iii)). A very high density of α-syn labeling was observed compared to dityrosine labeling (Fig. 1c(iii)), and this difference may suggest that some, but not all α-syn molecules contain dityrosine. Elevated levels of several metals have been reported in the substantia nigra of PD brains^15^,^16^,^19^, suggesting the mechanism by which dityrosine can be formed may be metal-induced. Therefore, Cu^2+^ was tested for the ability to induce the dityrosine cross-linked α-syn and the structural consequences of these dityrosine cross-links were investigated *in vitro*.

### Oxidation of α-syn enhances dityrosine cross-link formation

**Figure 2:**
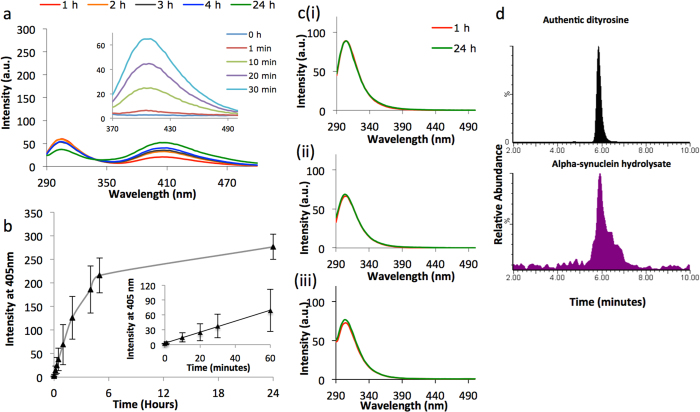
α-syn forms dityrosine cross-links in the presence of Cu^2+^/H_2_O_2_. (**a**) 50 μM of monomeric α-syn was incubated for 24 h with Cu^2+^/H_2_O_2_ at 37 °C and agitation of 400 rpm. A fluorescence spectra was collected with excitation wavelength of 280 nm to explore tyrosine and dityrosine signals. Fluorescence spectra was also collected using excitation wavelength of 320 nm to focus on dityrosine signals and to record the early time points (inset). After one hour of incubation, the tyrosine fluorescence signal appeared to decline with simultaneous appearance of a new increasing signal at 405–410 nm, typical of the dityrosine fluorophore. (**b**) shows the development of dityrosine signal (at 405 nm) (ex. 320 nm) over time for 24 h and over 1 hour (inset). (**c**) Using an excitation wavelength of 280 nm, no change in the tyrosine signal and an absence of dityrosine fluorescence signal was observed over 24 h of incubation of 50 μM α-syn alone (i), with 50 μM Cu^2+^ only (ii) or with 1.25 mM H_2_O_2_ only (iii). (**d**) LC-ESIMS/MS (MRM) was used to detect dityrosine in the oxidized α-syn hydrolysate. The oxidized α-syn was obtained by oxidation of (50 μM) monomeric α-syn for 24 h with Cu^2+^/H_2_O_2_. Data is shown in a chromatogram as relative abundance against retention time showing the dityrosine standard for comparison.

The formation of dityrosine cross-linked α-syn was enhanced by incubating 50 μM recombinant α-syn with Cu^2+^ and hydrogen peroxide at pH 7.4 and for 24 hours at 37 °C with agitation (Fig. 2a). Controls were prepared by incubating 50 μM recombinant α-syn in buffer alone or with buffer supplemented with Cu^2+^ or H_2_O_2_ only (Fig. 2c). The incubation of α-syn with Cu^2+^/H_2_O_2_ leads to rapid loss of the intrinsic tyrosine fluorescence signal (305 nm) concomitant with an enhancement in fluorescence emission intensity at wavelength of 405–410 nm, indicating dityrosine cross-link formation (Fig. 2a) whilst α-syn with buffer only (Fig. 2c(i)), Cu^2+^ only (Fig. 2c(ii)) or H_2_O_2_ only (Fig. 2c(iii)) showed no change in tyrosine or dityrosine fluorescence intensity over 24 hour incubation period. The assignment of the fluorescence to dityrosine was further confirmed using an excitation wavelength of 320 nm and emission at 405 nm (inset Fig. 2a) supported by published data obtained at a similar pH^17^ and showed increased dityrosine signal over 24 hours incubation (Fig. 2b). The formation of dityrosine is rapid, with a signal appearing within 10 mins and increasing over 1 hour (Fig. 2b inset). The Cu^2+^/H_2_O_2_ oxidized α-syn was quenched using EDTA after 24 hours, acid hydrolysed and LC-ESIMS/MS was acquired using MRM mode to further confirm the identity of the resulting dityrosine cross-link (Fig. 2d). The mass chromatogram of α-syn hydrolysate revealed a peak with retention time of 5.8 min, which corresponded to the retention time for authentic dityrosine (Fig. 2d).

### Characterizing the nature of the dityrosine cross-linked α-syn

Fluorescence spectra (Fig. 2a) showed that incubating α-syn under oxidative conditions rapidly induces dityrosine cross-links in α-syn and 1 h was enough to observe a dityrosine signal with high intensity. Because α-syn has four tyrosine residues located at 39, 125, 133, 136, this raises the possibility that α-syn oxidation can lead to the formation of both intra- and inter-molecular dityrosine cross-links.

**Figure 3:**
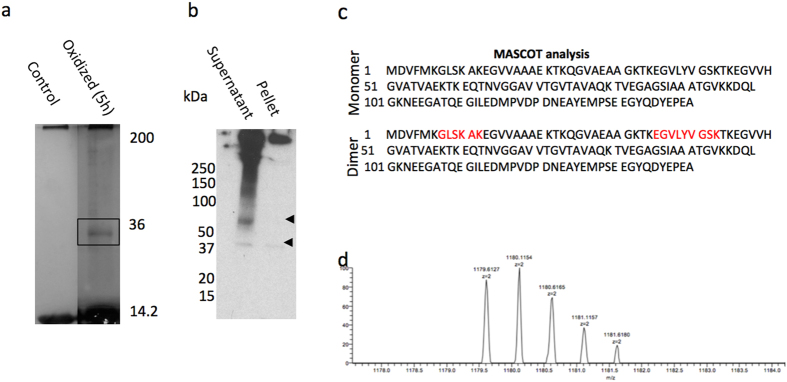
(**a**) SDS-PAGE analysis of α-syn samples. 100 μM of purified recombinant human α-syn was oxidized using Cu^2+^/H_2_O_2_ in 20 mM HEPES buffer, pH 7.4, at 37 °C with agitation of 400 rpm for 5 hours and EDTA was added to quench the oxidation process. SDS-sample buffer was added to the samples and then boiled for 5 min at 100 °C, and 10 μl of the boiled sample loaded on 12% Tris-glycine gel and then silver stained. The figure shows the appearance of a band with molecular weight about 35 kDa after 5 h oxidation indicating the presence of a dimer, which is not present in the non-oxidized control. (**b**) Shows western blot showing the separated pellet and supernatant labeled using the anti-dityrosine primary antibody. The antibody positive bands are highlighted by arrows which are likely to be dimer and tetramer in supernatant and only dimer and fibrillar species in the pellet. NanoLC-MS/MS analysis of tryptic digested α-syn monomer and dimer that were extracted from electrophoresis gel boxed in panel (a), (**c**) shows analysis from Mascot Engine. Sequence from the monomer band was complete, but two sequences were missing from dimer band in the oxidized sample, TKEGVL**Y**VGSK and GLSKAK (highlighted in red). (**d**) Mass spectrum of tryptic digested α-syn dimer showing the appearance of a peak at *m*/*z* 1179.6127 that corresponds to the dimer of the peptide with sequence of (_33_TKEGVL**Y**VGSK_43_) containing Y39.

Cu^2^H_2_O_2_ oxidized α-syn incubated for 5 h was analyzed by SDS gel electrophoresis and visualized by silver staining (Fig. 3a). Both oxidized and control samples displayed the presence of high levels of monomeric α-syn running at the expected molecular weight of 14.5 kDa. After five hours incubation under oxidation conditions α-syn also ran at a higher molecular weight band at around 35 kDa, which may represent covalently cross-linked dimer through tyrosine-tyrosine coupling. The molecular weight of this band is slightly higher than the expected 29–30 kDa and may be due to an altered conformation of the protein in SDS conditions^20^. This “dimer” band was not present in the non-oxidized control (Fig. 3a). To investigate whether the monomeric or dimeric α-syn contains dityrosine cross-links, Western blotting was conducted using the anti-dityrosine antibody. The α-syn samples were centrifuged to separate the fibril containing pellet from the soluble species in the supernatant. Figure 3b shows two bands at molecular weights 37 kDa and 70 kDa which are approximately consistent with dimer and tetramer in the supernatant and a dimer band in the pellet. Also both lanes show some high molecular weight species which are presumably protofibrillar and fibrillar structures. This result confirms that the dimeric species is dityrosine cross-linked and showing that this dimer can assemble further into tetramer and dityrosine containing fibrils, consistent with the observation that these dimers go on to form fibrillar structures.

#### Identification of dityrosine cross-linked α-syn dimer using nanoLC-MS/MS

To investigate which tyrosine residue(s) are involved in the formation of dityrosine cross-links, both intact oxidized and non-oxidized α-syn were analyzed using nanoLC-MS/MS from trypsin digested peptides extracted from the SDS PAGE gel (Fig. 3a). To identify the α-syn peptides, the Mascot search engine was used and the search results revealed 100% sequence recovery of monomeric α-syn and 89% of dimeric α-syn (Fig. 3c). Interestingly, in the dimer band sample two regions were not mapped, corresponding to _7_GLSKAK_12_ and _35_EGVLYVGSK_43_ (Fig. 3c). The reason for the missing 7–12 region is unclear, however, the second region contained Y39, and one explanation for the absence of this sequence is that Y39 is oxidized and as a result a dityrosine cross-link was formed. As dityrosine exhibits high protease resistance, this peptide fragment was not released and instead a dimeric form of the _35_EGVLYVGSK_43_ peptide is released, indicating the involvement of Y39 to form a homodimer α-syn. This was confirmed by the appearance of a peak at *m/z* 1179.6127 and identified to be a dimer peptide of (_33_TKEGVLYVGSK_43_) as shown in (Fig. 3d). The potential involvement of Y39 in the formation of a heterodimer, i.e. Y39-Y125, Y39-Y133 or Y136, was also investigated but the data showed no evidence of formation of side chain links. Furthermore, the Mascot data showed the presence of the regions that contained Y125, Y133, and Y136 in both monomeric and dimeric α-syn (Fig. 3c) indicating that the α-syn dimer results from cross-linking between Y39 from two molecules to form a dimer.

### α-syn conformation and amyloid formation in the presence of Cu^2+^ ions

**Figure 4:**
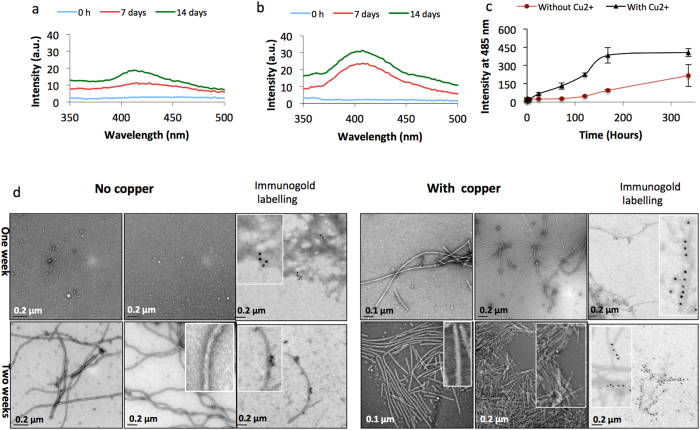
Slow oxidation of α-syn incubation in HEPES buffer only (**a**) and with Cu^2+^ (**b**). Incubation of 100 μM α-syn with or without Cu^2+^ only, showed the appearance of a dityrosine signal at 405 nm after 7 days of incubation which increases over 14 days. The presence of Cu^2+^ significantly increases dityrosine formation compared to buffer only. (**c**) ThT fluorescence assay to monitor fibril formation showed that the intensity increased significantly more for the α-syn incubated with 100 μM Cu^2+^ compared to α-syn without Cu^2+^ over 14 days. (**d**) TEM micrographs show comparison of α-syn incubated with and without Cu^2+^. The Cu^2+^ incubated α-syn assemblies display characteristic fibril morphology by negative stain TEM whilst sparse assemblies are observed for α-syn incubated without Cu^2+^. Immunogold labeling TEM for dityrosine cross-links reveals increased gold labels in α-syn incubated with Cu^2+^ and very few when Cu^2+^ is absent. Gold labels are evenly distributed along the fibrils (inserts).

At low concentration, α-syn does not self-assemble within a short time frame. Therefore, we examined dityrosine formation and assembly of α-syn by increasing the concentration to 100 μM. 100 μM α-syn incubated buffer alone (Fig. 4a) or with Cu^2+^ only (Fig. 4b), revealed the appearance of a fluorescence peak around 405 nm after 7 days of incubation which increased to 14 days. A higher intensity fluorescence signal was observed for those incubated with Cu^2+^ (Fig. 4b).

The samples were also monitored over two weeks using ThT fluorescence, negative-stain TEM and immunogold labeling for dityrosine. ThT fluorescence spectra of Cu^2+^-induced α-syn fibrils revealed a significant increase in intensity over the two week incubation period (Fig. 4c), revealing α-syn fibril formation after seven days of incubation, and this was further confirmed by observation of fibrils in TEM images (Fig. 4d). After one week of incubation, α-syn fibrils formed when incubated in the presence of Cu^2+^, whilst very few α-syn fibrils were detected by TEM or ThT fluorescence in the sample incubated under control conditions. The distribution of dityrosine was further examined with immunogold labeling using an anti-dityrosine antibody which revealed sparse distribution of dityrosine labeling on the buffer incubated α-syn fibrils (Fig. 4d) compared to a regular distribution of gold labels along the fibrils incubated in Cu^2+^ only (Fig. 4d). Also, the morphology of the fibrils differed whereby those incubated with Cu^2+^ were more plentiful and showed some lateral association or crossing over of straight, well ordered filaments compared to the more twisted fibrils formed in buffer alone.

**Figure 5:**
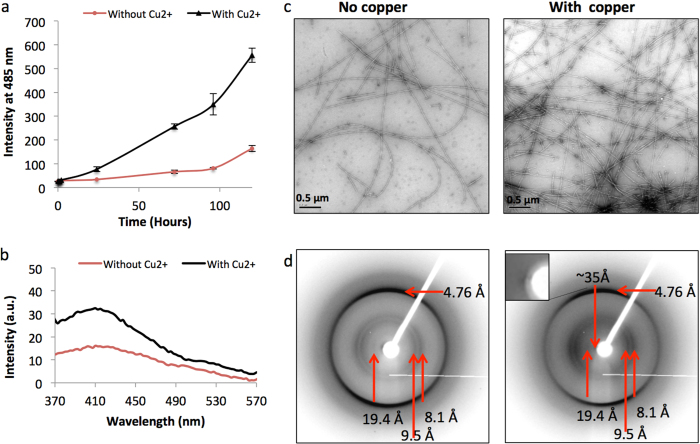
Cu^2+^ ions affect α-syn fibril growth and structure. 400 μM α-syn fibrils grown in 20 mM HEPES buffer, pH 7.4 with and without Cu^2+^ with agitation of 450 rpm. (**a**) ThT fluorescence assay over 120 hours revealed increased fluorescence for α-syn incubated with Cu^2+^ compared to α-syn without Cu^2+^. (**b**) Shows fluorescence at 405 nm following incubation of α-syn for 120 hours confirming that dityrosine forms in 400 μM α-syn and that Cu^2+^ enhances its formation. (**c**) TEM micrographs reveal no significant morphological changes between α-syn with or without Cu^2+^, although there appear to be increased number of fibrils in Cu^2+^ induced samples, consistent with the ThT results. (**d**) X-ray fibre diffraction patterns collected from partially aligned α-syn fibrils incubated at 400 μM α-syn without and with Cu^2+^ reveals the characteristic cross-β pattern. Inset shows a zoom of ~35 Å reflection close to the back stop.

In order to further explore the structure of dityrosine cross-linked α-syn, a high concentration of fibrillar α-syn was obtained by incubation of 400 μM monomeric recombinant human α-syn in the absence or presence of 400 μM Cu^2+^. ThT fluorescence assay confirmed the assembly of α-syn in the presence and absence of Cu^2+^ over 120 hours, and showed that the presence of Cu^2+^ appeared to enhance the rate of assembly (Fig. 5a). Fluoresence showed increased intensity at 405 nm in the Cu^2+^ samples confirming the presence of dityrosine (Fig. 5b). Electron micrographs of α-syn fibrils formed under control and Cu^2+^-containing buffer conditions after 120 h incubation (Fig. 5c) again revealed that incubation with Cu^2+^ markedly accelerates fibril formation (Fig. 5a). TEM micrographs revealed a higher density of Cu^2+^-enhanced α-syn fibrils that also appeared to have a more ordered, rigid appearance (Fig. 5c). This provided further evidence that the Cu^2+^ ion plays a role in accelerating α-syn fibril formation and may affect the final structure.

To verify that the samples contained the expected cross-β amyloid fibrils, XRFD patterns were collected for both α-syn fibrils incubated with Cu^2+^ ions and those assembled without Cu^2+^ ions. The XRFD patterns showed the expected amyloid cross-β pattern^21^,^22^,^23^, for both Cu^2+^-enhanced α-syn fibrils and control α-syn fibrils (Fig. 5d). The expected cross-β meridional reflections at 4.76 and 2.4 Å were observed in patterns from fibrils assembled under both control (i.e. without Cu^2+^) and Cu^2+^-containing conditions. Moreover, a set of reflections at ~8.1, 9.5 and 19.4 Å were observed on the equator, similar to those reported in a previous study^24^, confirming the formation of α-syn fibrils with cross-β structure. The equatorial diffraction signals arise from structural distances perpendicular to the fibre axis, such as sheet spacing, protofilament packing and protofilament size. However, the XRFD for Cu^2+^-enhanced α-syn fibrils (Fig. 5d) revealed a new reflection at ~35 Å that may arise from regular lateral packing of protofilaments and also showed more intense reflections attributable to the formation of more aligned fibrils compared to those assemblies incubated under control conditions.

### Morphology and stability of dityrosine cross-linked α-syn

**Figure 6:**
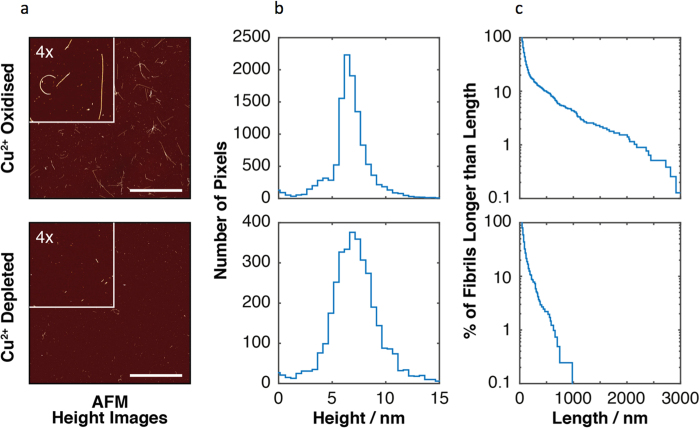
Quantitative AFM imaging analysis of α-syn fibrils grown for 2 weeks in Cu^2+^ oxidized or Cu^2+^ depleted conditions (using EDTA). (**a**) AFM height images of α-syn fibril samples formed in Cu^2+^ oxidized and Cu^2+^ depleted conditions, respectively. The scale bars indicate the length of 10 μm. Magnified (4X) images are shown as insets. (**b**) Height distributions of the pixels of the fibril particles are shown as histograms, indicating the width of the fibrils formed in Cu^2+^ oxidized and Cu^2+^ depleted conditions, respectively. (**c**) Length distributions of fibril particles formed in Cu^2+^ oxidized and Cu^2+^ depleted conditions, respectively. The length distributions are show as 1-cumulative distribution functions on a semi-log plot to enable visual comparison for the wide length distributions of the samples. A total of 781 fibrils formed in Cu^2+^ oxidized conditions and 409 fibrils formed in Cu^2+^ depleted conditions were analyzed. 100 μM α-syn shaken for 2 weeks at 37 °C with 100 μM Cu^2+^ or with EDTA (2.5 mM) was diluted to 2 μM for AFM imaging.

To compare α-syn fibrils containing cross-links with those without cross-links, α-syn was allowed to self-assemble for two weeks in the presence of Cu^2+^ or EDTA was used to deplete the Cu^2+^. Cu^2+^ depleted α-syn was confirmed to be lacking dityrosine crosslinks from fluorescence assays (data not shown). Following mechanical stirring to disrupt the preformed fibrils, the structure and morphologies of the α-syn fibrils formed in Cu^2+^ oxidizing and Cu^2+^ depleted environments were next analyzed using AFM. Height images of both Cu^2+^ oxidized and Cu^2+^ depleted fibrils deposited on mica surfaces were acquired over 30 × 30 μm surface areas imaged at resolution of 2048 × 2048 pixels for qualitative analysis (Fig. 6a). The height distribution of the pixels of the fibril particles extracted from the AFM images are shown in Fig. 6b. α-syn fibrils grown in the presence of Cu^2+^ often appeared to be linked, crossed-over or attached to form star shaped structures, whilst those grown without Cu^2+^ appeared shorter and more dispersed. The average height 6.6 ± 2.0 nm SD and 7.1 ± 2.3 nm SD for fibrils grown in Cu^2+^ oxidizing and Cu^2+^ depleted environment, respectively, suggests that aggregation in both a Cu^2+^ oxidizing and Cu^2+^ depleted environment results in fibrils of similar morphologies and width. However, the extracted contour length distributions for the fibril samples (Fig. 6c), displayed an increased average length for fibrils formed in a Cu^2+^ oxidizing environment compared with fibrils formed in Cu^2+^ depleted conditions. This is consistent with the idea that Cu^2+^ mediates enhancement of aggregation through stabilization of the aggregates. Fibrils formed in a Cu^2+^ depleted environment display a weighted average contour length of 220 ± 10 nm SE. This is considerably shorter than fibrils formed in Cu^2+^ oxidized conditions, which present a weighted average contour length of 880 ± 30 nm SE. This increased fibril length of fibrils formed in a Cu^2+^ oxidized enviroment supports the formation of dityrosine cross-linked dimers as a critical step in the early stages of α-syn aggregation. The ability of α-syn to form fibrils of increased average length in the presence of Cu^2+^, therefore, further supports an important role of dityrosine cross-linked dimers in the formation of amyloid assemblies, as the presence of additional core interactions provided by the dityrosine cross-links may responsible for an increase in the stabilization of the amyloid core and accelerated amyloid assembly.

**Figure 7:**
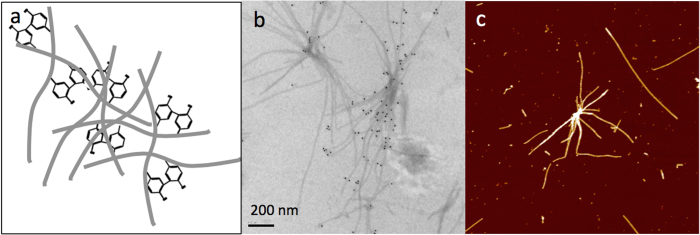
Dityrosine cross-links form between fibrils to generate intertwined and interconnected structures. (**a**) Schematic showing fibrils connected via dityrosine cross-links, (**b**) electron micrograph showing dityrosine immunogold labeling of fibrils and (**c**) AFM image showing interconnected fibrils. α-syn fibrils were formed using 100 uM α-syn incubated with 100 uM Cu^2+^ in 20 mM HEPES buffer at pH 7.4 with 400 rpm agitation for 30 days incubation.

*In vitro* growth of α-syn in the presence of Cu^2+^ was monitored after prolonged incubation using electron microscopy (Fig. 7a) and atomic force microscopy (Fig. 7c) and reveals the formation of networks and star-like structures. We propose that these structures may form the basis for the distinctive Lewy body shape (Fig. 7).

## Discussion

PD is the second most common neurodegenerative disease after AD^25^. Cu^2+^ has been found to decrease in the substantia nigra and increase in the CSF of PD patients^26^,^27^. Oxidative stress is implicated in the pathogenesis of PD and modification of tyrosine residues may play a role in the α-syn aggregation via protein cross-linking. We have revealed that Lewy bodies in PD patient brain tissues contain α-syn and dityrosine cross-links. These structures may serve to allow the fibrils within Lewy body deposits to persist. Furthermore, the formation of dityrosine cross-linked dimers may play an important role in oligomer toxicity in animal models^4^. Dityrosine cross-linked α-syn oligomers generated using PICUP using tris(bipyridine) ruthenium(II) chloride complex as a photosensitizer have been shown to exhibit high toxicity on differentiated neuronal-like SH-SY5Y cells, indicating the importance of the tyrosine residue oxidative modification in the etiology of PD^17^. Previous work has revealed that disordered, soluble α-syn can persist in mammalian cells and Y39 was identified as playing a role in maintaining a compact structure^28^.

To further characterize the role of the dityrosine cross-links in self-assembly and structure, α-syn was examined *in vitro* using fluorescence spectroscopy, LC-ESIMS/MS, TEM, XRFD and AFM following incubation in oxidative conditions and with Cu^2+^. We showed that the rate of assembly was enhanced for α-syn incubated with Cu^2+^. This was further confirmed by electron micrographs showing increased formation of α-syn fibrils in the presence of Cu^2+^ compared with control, which shows the accumulation of amorphous or spherical aggregates of α-syn after one week and fibrils forming after 2 weeks. Formation of dityrosine, detected using fluorescence, showed an increased intensity after 7 days incubation for α-syn with Cu^2+^. α-syn without Cu^2+^ was also able to form dityrosine, but at a much slower rate. This indicates a role for metal catalyzed oxidation (MCO) in initiating the α-syn amyloid formation process *in vitro*, and may implicate a metal catalyzed mechanism *in vivo*. AFM results reveal that the dityrosine cross-links may also stabilize the amyloid core resulting in accelerated assembly and thereby fibrils of longer contour lengths. It has been shown previously that dityrosine forms in α-syn fibrils that have been aged in the absence and presence of Cu^2+^ in an aerobic environment, whilst α-syn aged anaerobically did not form dityrosine cross-links^29^. Furthermore, incubation of α-syn in solutions that contain methionine and/or EDTA, which help to prevent or reduce the level of protein oxidation, led to lengthened lag times for fibril formation^18^ and here reduced the length of α-syn fibrils revealed by AFM. The results show that α-asyn is able to assemble in the absence of dityrosine cross-link formation but that the assembly is slowed. It has been shown previously that stable α-syn polymers can be generated using nitrating agents and these polymers are stabilized due to the formation of dityrosine cross-link^30^. Collectively our results indicate that Cu^2+^ ions strongly promote the oxidation of α-syn, resulting in dityrosine cross-linked α-syn.

It has been found that seeding α-syn with dityrosine cross-linked dimers accelerates α-syn fibril growth, suggesting that the critical rate-limiting step in the nucleation of α-syn fibrils is the formation of dityrosine cross-linked dimeric species^18^. Here, a covalently cross-linked dimer was isolated by SDS-PAGE and confirmed to be dityrosine by western blotting and using nanoLC-MS/MS following tryptic digestion. The mass analysis showed that dityrosine dimer was formed via the coupling of Y39-Y39 to give a homodimer. This observation is supported by previous work showing that a α-syn mutant Y125W/Y133F/Y136F where a single Y remained at position 39 showed the same ability to form fibrils as wild type α-syn^31^, reflecting the important role played by Y39 in the α-syn fibrillation. Quenched hydrogen/deuterium exchange NMR spectroscopy was used previously to study the structure of α-syn in its amyloid state and identified five β-strands within the fibril core, comprising residues 35–96 and termed aSβ1, aSβ2, aSβ3, aSβ4 and aSβ5^32^. Tyrosine at position 39 is found within aSβ1, while tyrosine residues at 125, 133, and 136 did not contribute towards the formation of any β-strand. Recently, the first region consisting of _37_VLYVGSKT_44_ was studied using XRFD and it was found that at high concentrations the peptide forms nanotubular cross-β assemblies^33^. Interestingly, the proposed structural model of these nanotubes revealed that peptide packing and inter-sheet Tyr interactions were involved to stabilize the tape width^33^. Also, the model shows that the orientation of the phenol groups of Tyr residues is in agreement with the formation of inter-sheet dityrosine cross-links, however, it is important to keep in mind this may be not be true for fibrils formed by full length α-syn. Indeed, a very recent structure of the α-syn fibril elucidated using solid state NMR combined with X-ray fibre diffraction revealed that residues 44–96 form the core of the fibril. Residue 39 in this structural context is found at the edge of the structured core and within a disordered region and therefore is available for dityrosine cross-linking between and within fibrils^34^.

These results are in good agreement with other published data^18^,^29^,^30^, and support the notion that dityrosine cross-linked α-syn oligomers can serve as seeds in the α-syn amyloid formation process and are able to stabilize the amyloid oligomers and fibrils. This may be important in the formation of Lewy bodies which persist in PD brain.

### Concluding remarks

Many studies have demonstrated that dityrosine cross-linked α-syn species reduce the yield of amyloid fibrils^14^,^17^, while dityrosine formation has been described to be the critical rate-limiting step in α-syn assembly^18^. These results appear to be in conflict and this could be interpreted by the variation of the *in vitro* oxidative stress conditions that have been used to form the dityrosine cross-links. Also, there are many factors that can affect the lag duration such as α-syn concentration, buffer composition and pH, and temperature^35^,^36^,^37^. In order to gain better understanding of the relationship between dityrosine oligomer formation and α-syn assembly, we have used *in vitro* oxidative stress conditions that mimic the *in vivo* conditions and revealed dityrosine cross-linked α-syn *in vivo* in Lewy bodies in PD brain.

## Materials and Methods

### Immunogold Labeling TEM of tissue sections

PD brain tissue from substantia nigra was obtained from Parkinson’s disease UK Brain Bank. Tissue was removed according to Local Ethics Committee guidelines, and informed consent for brain donation was obtained from the next of kin and stored at -80 °C until required (Table 1).

Brain tissue blocks were prepared for immunogold labeling TEM by minimal cold fixation and embedding protocols, as previously described^38^. Immunogold labeling was performed using an established methodology^39^, with PBS+ buffer being used for all dilutions of immunoreagents and for rinsing. A modified phosphate-buffered saline, pH 8.2, containing 1% BSA, 500 μl/l Tween-20, 10 mM Na EDTA, and 0.2 g/l NaN3 (henceforward termed PBS+), was used throughout all the following procedures for all dilutions of antibodies and secondary gold probes. Thin sections were collected upon TEM support grids, then incubated with normal goat serum (1.10 dilution) for 30 min at room temperature to block non-specific secondary antibody binding. Grids were then labeled with (10 μg/ml IgG) anti-dityrosine mouse monoclonal antibody (Japan Institute for the Control of Aging JaICA, Shizuoka, Japan) or double-labeled using a mixture of (10 μg/ml IgG) anti-α-syn (C-20)-R rabbit polyclonal antibody (Santa Cruz Biotechnology, Inc.) and (10 μg/ml IgG) anti-dityrosine mouse monoclonal antibody and incubated overnight at 4 °C. After 3 × 2 min PBS+ rinses, sections were then immunolabeled with GaM10 (10 nm diameter) or a mixture of GaR5 (5 nm diameter) and GaM15 (15 nm diameter) secondary probes (all 1.10 dilution), for 1 h at room temperature. After 3 × 10 min PBS+ and 4 × 5 min distilled water rinses, the grids were post-stained in 0.22 μM-filtered 0.5% (w/v) aqueous uranyl acetate for 1 h. The grids were examined on a Hitachi 7100 TEM (Hitachi, Germany) fitted with a Gatan Ultrascan 1000 CCD camera (Gatan, Abingdon, UK), and operating with a voltage of 100 kV.

### Synthesis of a dityrosine standard

A dityrosine standard was synthesized using horseradish peroxidase and *N*-acetyl-3,5-diiodo-L-tyrosine as a starting material as described previously^9^.

### Preparation of α-syn

The lyophilized full-length human recombinant α-syn (1 mg) in final buffer of 10 mM Tris-HCl (pH 7.4) was purchased from rPeptide (Bogart, GA, USA) or prepared as previously described^40^. α-syn was of high purity and does not show any cysteine misincorporation to tyrosine at position 136, which has been observed for about 20% of human α-syn expressed in *Escherichia coli*^41^. The peptide was resuspended in Milli-Q filtered water at concentration of 2 mg/ml, and then the Tris-HCl buffer was removed using Vivaspin-500 concentrator tube, 3000 MWCO PES (Sartorius stedim biotech, Germany). Briefly, the resuspended α-syn was centrifuged at 14,000 g for 30 min, subsequently the obtained concentrated α-syn was again diluted with Milli-Q filtered water at a concentration of 2 mg/ml and the concentrating process was repeated. The resulting α-syn solution in water was collected and filtered using 0.22 μm syringe filter to remove any preformed aggregates. Finally, the concentration was determined using a molar extinction coefficient of 5120 M^−1^cm^−1^ and the absorbance was measured at a wavelength of 280 nm using an Eppendorf Biophotometer (Eppendorf UKLtd., Cambridge, UK)^29^.

### Cu^2+^- catalyzed oxidation of α-syn

Prior to oxidation, α-syn stock solution was prepared as described above, to remove any preformed aggregates and fibrils. Soluble α-syn monomer was incubated with Cu^2+^ using molar ratio of 1.1 and concentration of 50 μM. To initiate the oxidation reaction, H_2_O_2_ (1.25 mM) was added. The oxidation reaction was performed in 20 mM HEPES (pH 7.4) at 37 °C with agitation of 400 rpm. After 24 h, the oxidation reaction was stopped using EDTA at a final concentration of 1.25 mM. The dityrosine formation was monitored using a fluorescence spectrophotometer (Varian Ltd., Oxford, UK). Controls were obtained by incubation of 50 μM α-syn alone, with H_2_O_2_ (1.25 mM) only and with Cu^2+^ only in 20 mM HEPES buffer pH 7.4 at 37 °C with agitation of 400 rpm.

### Exploring the effect of Cu^2+^ on the α-syn fibrillogenesis and structure

To examine the slow oxidizing effect of Cu^2+^ only on the α-syn fibrillogenesis, two concentrations of α-syn (100 and 400 μM) were examined, whilst maintaining a molar ratio of 1.1 with Cu^2+^. The samples were then further analyzed as described below.

### Fluorescence Spectroscopy

The fibrils were resuspended by agitation and fluorescence spectra were collected at time intervals. Fluorescence measurements were carried out on a Varian Cary Eclipse fluorimeter (Varian Ltd., Oxford, UK) using a 1 cm path length quartz cuvette (Starna, Essex, UK), and dityrosine fluorescence was monitored using an excitation wavelength of 320 nm. Dityrosine emission was monitored between 340 and 500 nm, with maximum fluorescence intensity at around 405–410 nm at a controlled temperature of 21 °C. To detect dityrosine fluorescence at early time points, 130 μl of the reaction mixture was removed and EDTA was added to final concentration of 1.25 mM.

Tyrosine fluorescence signal was monitored using an excitation wavelength of 280 nm and emission wavelength of 305 nm. Excitation and emission slits were both set to 10 nm, and the scan rate was set to 300 nm/min with 2.5 nm data intervals and an averaging time of 0.5 s. The photomultiplier tube detector voltage was set at 500 V.

### Sample preparation for LC-ESIMS/MS analysis

For LC-ESIMS/MS analysis, 24 h incubated, oxidized α-syn (50 μM) was first desalted to remove the HEPES buffer that could affect the mass spectrum. The desalting was performed using Amicon ultra-4 centrifugal filter units, 10 k NMWL (M Millipore, USA) and centrifuged at 3900 g and a temperature of 4 °C. The resulting α-syn was then hydrolyzed using evacuated sealed tubes under acidic conditions of (6 M) HCl, 10% TFA, and 1% phenol at 110 °C for 24 h. The resulting hydrolysate was then dried under nitrogen gas, dissolved in 100 μl of 0.1% formic acid in water and then filtered using a Millipore 0.22 μm filter into a 0.2 ml tube.

### Detection of Dityrosine by LC-ESIMS/MS

20 μl of oxidized α-syn hydrolysate was injected on to a Phenomenex Gemini 3 u C_6_-phenyl 110 (150 mm × 4.6 mm, 3 micron) column using a high performance liquid chromatography (HPLC) system (Waters Alliance 2695, Ireland) coupled to the mass spectrometer (MicroMass Quattro Premier, Waters, Ireland) operated in the multiple reaction-monitoring (MRM) mode with positive electrospray ionization (ESI). The solvents for the mobile phase were A. 0.1% formic acid in water; and solvent B. 0.1% formic acid in acetonitrile. The gradients were as follows. t = 0 min, 0% B; t = 1 min, 0% B; t = 15 min, 100% B; t = 20 min, 100% B; t = 25 min, 0% B; t = 30 min, 0% B, and the flow rate was 200 μl/min. Mass spectrometric detection was performed by positive electrospray ionization (ESI) tandem mass spectrometry on a triple quadrupole mass spectrometer (MicroMass Quattro Premier, Waters, Ireland). The conditions for the mass spectrometer were as follows; electrospray ionization spray voltage 3.5 kV, the cone voltage 35 V, the source temperature at 100 °C, whereas the desolvation temperature was 400 °C. Argon was used as the collision gas at 5.95 e^−^003 mbar at 26 ev collision energy.

### Thioflavin T fluorescence assay

The fibril formation of α-syn was monitored using ThT fluorescence. A 0.2 μm filtered (3.14 mM) aqueous ThT stock solution was prepared and stored frozen at −20 °C in 1–10 μl aliquots until required. ThT was added to a 10 μM α-syn sample (20 mM HEPES buffer pH 7.4) to a final concentration of 20 μM, and then EDTA was added to a final concentration of 250 μM. The resulting mixture was then gently vortexed, and allowed to bind for 3 minutes before a reading was taken. Using a microvolume cuvette of 1 cm path length, ThT fluorescence was measured using a Varian Cary Eclipse fluorimeter (Varian, Oxford, UK) with excitation wavelength of 450 nm. The emission spectrum was recorded between 460–600 nm at 21 °C. HEPES buffer baselines were subtracted from the data. Excitation and emission slits were set to 5 nm and 10 nm respectively. The scan rate was 600 nm/min with 1 nm data intervals and an averaging time of 0.1 s. The voltage on the photomultiplier tube was set to high (800 v) and experiments were carried out in triplicate to confirm trends.

### Immunogold labeling, negative stain TEM for fibrils

The α-syn fibrils were immunogold-labeled ‘on grid’ for dityrosine^9^ using a monoclonal anti-dityrosine antibody (JaICA, cat. no. MDT-020P). The antibody has been fully characterized and shown to be highly specific and does not show any cross-reactivity with other tyrosine derivatives such as nitrotyrosine, chlorotyrosine^42^. The specificity and the antibody were also checked in our previous work using the identical procedure and IgG concentrations, with an irrelevant antibody to hair cell antigen (MAb10)^9^.

The antibody will detect any protein containing dityrosine. Briefly, 4 μl aliquots of the α-syn fibrils were pipetted onto Formvar/carbon coated 400 mesh copper TEM support grids (Agar Scientific, Essex, UK), left for 1 min, the excess was removed by filter paper, and then blocked in normal goat serum (1.10 in PBS+) for 15 min. Grids were then incubated with (10 μg/ml IgG) mouse dityrosine monoclonal antibody (JaICA, Shizuoka, Japan) for 2 h at room temperature, rinsed in 3×2 min PBS+, and then immunolabeled in a 10 nm gold particle-conjugated goat anti-mouse IgG secondary probe (GaM10 British BioCell International, Cardiff, UK; 1.10 dilution) for 1 h at room temperature. After 5 × 2 min PBS+ and 5 × 2 min distilled water rinses, the grids were negatively stained as described in negative stain TEM methods below.

### Negative stain TEM

Four μl aliquots of α-syn samples were placed onto Formvar/carbon coated 400-mesh copper grids (Agar Scientific, Essex, UK) for 1 min, and the excess was removed using filter paper. Subsequently the grid was washed using 4 μl of Milli-Q water filtered with 0.22 μM filter and blotted dry, then negatively stained twice with 4 μl of filtered 2% (w/v) uranyl acetate for 1 min and blotted dry. The grid was allowed to air-dry before examination on a Hitachi 7100 transmission electron microscope (Hitachi, Germany) fitted with a Gatan Ultrascan 1000 CCD camera (Gatan, Abingdon, UK) at an operating voltage of 100 kV.

### SDS gel electrophoresis

The separation gel consisted of 12% (w/v) acrylamide, 0.32% (w/v) bis-acrylamide, 0.1% SDS, and 0.38 M Tris-HCl (pH 8.8). The polymerization was initiated by addition of 5 μl TEMED and 50 μl freshly prepared ammonium persulfate (APS) solution per 10 ml of the gel solution. The stacking gel consisted of 5% (w/v) acrylamide, 0.13% (w/v) bis-acrylamide, 0.1% (w/v) SDS, and 0.38 M Tris-HCl (pH 6.8) and polymerization was initiated by addition of 5 μl TEMED and 20 μl freshly prepared ammonium persulfate (10% w/v) per 4 ml of the gel solution. Electrophoresis buffer consisted of 25 mM Tris-HCl (pH 8.3), 19 mM glycine, and 0.1% (w/v) SDS. Laemmli sample buffer (Sigma-Aldrich, UK), which consisted of 4% SDS, 20% glycerol, 10% 2-mercaptoethanol, 0.004% bromophenol blue and 0.125 M Tris- HCl (pH 6.8), was used in preparation of the protein samples using 1.1 volume ratio, then the resulting mixture was boiled for 5 min at 100 °C and centrifuged for 1 min. The SDS electrophoresis analysis was performed using Bio-Rad chamber (Bio-Rad, Hercules) under constant voltage (200 V). A protein marker standard (Sigma-Aldrich, UK) with range of 6,500–200,000 Da was run alongside the samples.

The separated protein bands were visualized using Silver staining. The silver staining was performed according to the manufacture’s instructions (Bio-Rad, U.S.).

### Western blotting

α-synuclein was oxidized using 100 μM alpha-syn with 100 μM Cu^2+^ in 20 mM HEPES buffer at pH 7.4 and incubated at 37 °C with 400 rpm agitation for 30 days. Samples were centrifuged at 14,000 g at 4 °C for 20 minutes to separate the supernatant and pellet. The concentration of the pellet was estimated by subtracting the concentration of protein in the supernatant from the total concentration using a molar extinction coefficient of 5120 M^−1^cm^−1^ and the absorbance was measured at a wavelength of 280 nm using an Eppendorf Biophotometer (Eppendorf UK Ltd., Cambridge, UK)^29^. An aliquot of 5 μg of protein of the sample was mixed with 4x Laemmli sample buffer (containing 2-mercaptoethanol) and heated to 95 °C for 5 mins. Samples were loaded and run on SDS-PAGE (4-20%Tris-Glycine gel (Bio-Rad)) and transferred to PVDF membrane, blocked with 5% milk (w/v) in Tris-buffered saline, 0.1% Tween 20 (TBST) and then probed with anti-dityrosine (DT) monoclonal antibody (IC3; JaICA diluted 1/1000) overnight at 4 °C. The blot was incubated with an anti-mouse HRP conjugate (7076 S from Cell Signaling diluted 1/1000) for 1 hour at room temperature and developed with ECL reagent (Bio-Rad) following the manufacturer’s instructions.

### In gel digestion protocol

The α-syn oxidation mixture (after 5 h of oxidation) were separated by SDS-PAGE and stained using Coomassie Blue, then the separated bands (monomer and dimer) were excised and divided into 3–4 pieces and de-stained with 30% acetonitrile for 15 min with agitation followed by 50% acetonitrile/25 mM ammonium bicarbonate for 15 min with agitation. This step was repeated until the gel pieces were completely de-stained. The de-stained gel pieces were then dehydrated using vacuum centrifugation for 5 min without heating. 12.5 ng/ml of trypsin solution in 25 mM ammonium bicarbonate was added and the gel pieces were allowed to rehydrate for 5 min, then the excess trypsin solution was removed and the gel pieces covered by 25 mM ammonium bicarbonate and incubated at 37 °C for 6 h. Formic acid was added to ~5% (v/v) and the resulting mixture was vortexed and centrifuged at 1000 g for 1 min and the supernatant was removed, the remaining peptides were extracted from the gel pieces using 50% acetonitrile with agitation and brief sonication. This step was repeated and all supernatants were pooled and the volume was reduced by vacuum centrifugation. The resultant sample solution was stored at −80 °C until analyzed by nanoLC-LTQ-OrbitrapXL mass spectrometry.

### α-syn fibril preparation for X-ray fibre diffraction

α-syn fibrils were formed in 20 mM HEPES buffer (pH 7.4) by incubation with and without Cu^2+^ at molar ratio of 1.1 and concentration of 400 μM for 5 days at 37 °C and with agitation of 450 rpm. To remove HEPES buffer, which can affect the quality of X-ray fibre diffraction data, the resulting fibrils were centrifuged for 30 min at 20,000 g and 4 °C, then the pellet was re-suspended in 10 μl Milli-Q water and aligned as described below.

### X-ray fibre diffraction (XRFD)

Fibre diffraction specimens were prepared by suspending a 10 μl droplet of α-syn fibril solution, which is prepared as described above, between two wax-tipped 1.2 mm O.D, 0.94 mm I.D borosilicate capillaries (Harvard apparatus), then left at room temperature in a parafilm sealed petri dish until dry. X-ray diffraction patterns were collected using a Rigaku 007HF CuKα (λ 1.5419 Å) rotating anode generator with a Saturn 944+ CCD detector with exposure times of 10–120 seconds and specimen to detector distances of 50 or 100 mm. The images were displayed and examined using Mosflm^43^.

### Atomic force microscopy AFM

α-syn stock solution was prepared as described above, to remove any preformed aggregates and fibrils. Soluble α-syn monomer was incubated with Cu^2+^ using a molar ratio of 1.1 and concentration of 100 μM in 20 mM HEPES (pH 7.4) at 37 °C with agitation of 400 rpm for two weeks. To compare between dityrosine crosslinked and non-crosslinked α-syn, dityrosine formation was prevented by depleting Cu^2+^ by adding EDTA (final concentration 2.5 mM) to 100 μM α-syn in 20 mM HEPES buffer pH 7.4 and incubating at 37 °C with agitation of 400 rpm for two weeks.

To prepare the samples for AFM, the fibril stock was diluted to 2 μM with milliQ water. Samples were then stirred at 1000 rpm for 1 hour in 1.5-ml glass vials containing 38 mm polytetrafluoroethylene-coated magnetic stirring bars to cause mechanical distruption. 20 μl of the samples were subsequently deposited on freshly cleaved mica and allowed to incubate for 8 minutes. The mica surfaces were then washed with a 1 ml filter sterilized milli-Q water, and dried using filter paper to absorb residual water followed by gentle stream of nitrogen gas. The samples were imaged with a Bruker multimode 8 scanning probe microscope operating under ScanAsyst peak force tapping mode equipped with SCANASYST-AIR, silicon nitride cantilever probes with a nominal tip radius of 2 nm and nominal spring constant of 0.4 N/m (Bruker Corporation, Massachusetts). Images were collected at a resolution of 2048 × 2048 pixels at a scan size of 30 × 30 μm and a scan rate of 0.4 Hz. The Images were processed using Nanoscope analysis software (version 1.4, Bruker Corporation, Massachusetts) to flatten and thereby removing surface tilt and bow on the images. Height and contour length was quantified in the AFM images using an in-house Matlab script that traces individual fibril particles^44^.

## Declarations

### Ethics

Ethics approval was obtained locally for this work (University of Sussex) and the tissue was obtained from Parkinson’s Disease UK (PDUK) brain bank under a Material transfer agreement. Informed consent is sought by PDUK.

## Additional Information

**How to cite this article**: Al-Hilaly, Y. K. *et al*. The involvement of dityrosine crosslinking in α-synuclein assembly and deposition in Lewy Bodies in Parkinson’s disease. *Sci. Rep.* **6**, 39171; doi: 10.1038/srep39171 (2016).

**Publisher's note:** Springer Nature remains neutral with regard to jurisdictional claims in published maps and institutional affiliations.

## Figures and Tables

**Table 1:** PD tissue (substantia nigra).

Case	Gender	Age/(years)	Duration/(years)	Post-mortem interval/(hours)
PD017	M	72	18	22
PD0028	M	82	18	14
PD0041	M	77	10	6
